# Supplementary material for: Detection of IDH1 Mutations in Plasma Using BEAMing Technology in Patients with Gliomas
Source: Cancers (Basel). 2022 Jun 11;14(12):2891. doi: 10.3390/cancers14122891 (PMC9221506; doi:10.3390/cancers14122891)
Supplement: Supplementary file 1 [file cancers-14-02891-s001.zip › SUPPLEMENTARY MATERIAL/FIGURE S1 CHRONOGRAM .pdf]

CHRONOGRAM OF PERIPHERAL BLOOD COLLECTION FOR IDH MUTATION DETECTION IN PLASMA													
Perioperative		RT +/- CT		Adjuvant CT						Follow-up			
Pre-SX	Post-SX	1st intraRT	2nd intraRT	Pre-CT	After 2nd cycle	After 3rd cycle	After 4th cycle	After 5th cycle	After 6th cycle	1st F-U	2nd F-U	3rd F-U	4th F-U
(-7d to -1d)	(+1wk to +6wk)	+ 2wk intraRT	+ 4wk intraRT	(-7d to -1d)	(-7d to -1d)	(-7d to -1d)	(-7d to -1d)	(-7d to -1d)	(-7d to -1d)	+4-12wk	+4-12wk	+4-12wk	+4-12wk
1	2	3	4	5	6	7	8	9	10	11	12	13	14
